# Sulphur Atoms from Methionines Interacting with Aromatic Residues Are Less Prone to Oxidation

**DOI:** 10.1038/srep16955

**Published:** 2015-11-24

**Authors:** Juan C. Aledo, Francisco R. Cantón, Francisco J. Veredas

**Affiliations:** 1 Departamento de Biología Molecular y Bioquímica, Facultad de Ciencias, Universidad de Málaga, 29071-Málaga, Spain; 2 Departamento de Lenguajes y Ciencias de la Computación, Universidad de Málaga, 29071-Málaga, Spain

## Abstract

Methionine residues exhibit different degrees of susceptibility to oxidation. Although solvent accessibility is a relevant factor, oxidation at particular sites cannot be unequivocally explained by accessibility alone. To explore other possible structural determinants, we assembled different sets of oxidation-sensitive and oxidation-resistant methionines contained in human proteins. Comparisons of the proteins containing oxidized methionines with all proteins in the human proteome led to the conclusion that the former exhibit a significantly higher mean value of methionine content than the latter. Within a given protein, an examination of the sequence surrounding the non-oxidized methionine revealed a preference for neighbouring tyrosine and tryptophan residues, but not for phenylalanine residues. However, because the interaction between sulphur atoms and aromatic residues has been reported to be important for the stabilization of protein structure, we carried out an analysis of the spatial interatomic distances between methionines and aromatic residues, including phenylalanine. The results of these analyses uncovered a new determinant for methionine oxidation: the S-aromatic motif, which decreases the reactivity of the involved sulphur towards oxidants.

The simple covalent addition of an oxygen atom to a sulphur atom of a methionine residue can change the physicochemical properties of the whole protein. Thus, the oxidation of methionine residues to methionine sulfoxide (MetO) both *in vitro* and *in vivo* has been reported to have multiple, varied implications for protein function.

*In vitro*, avoiding methionine oxidation is an important challenge for the pharmaceutical industry^1^. A common problem affecting therapeutic proteins in aqueous solution is related to the inactivating oxidation reactions that occur during all steps of the production procedure and throughout the shelf-life of the product^2^. Therefore, it is important to understand the factors influencing methionine vulnerability to oxidation in proteins. In this context, many studies have been devoted to characterizing the reactivity of methionyl residues from diverse protein pharmaceuticals^3^,^4^,^5^,^6^,^7^. Although accessibility of the sulphur atom to the solvent is an important factor, evidence from the literature has suggested that the solvent-accessible area is not sufficient to explain the disparate reactivities among methionine residues^4^,^8^,^9^. Therefore, the structural properties that modulate the reactivity of methionyl residues have remained unclear.

*In vivo*, a myriad of pathophysiological conditions including ageing^10^, neurodegenerative diseases^11^,^12^, pulmonary diseases^13^ and vascular diseases^14^,^15^ have been related to methionine modification. In addition, redox reactions involving methionyl residues have been proposed to be reversible covalent modifications with dynamic regulatory functions^16^,^17^,^18^. Interestingly, in the past several years, enzymes able to catalyse the reversible interconversion of methionine and MetO have been described^19^,^20^,^21^,^22^. Indeed, over the past two decades, research from a number of laboratories has helped to change the view of methionine as an unimportant proteinogenic amino acid that is readily interchangeable with other residues such as valine or leucine. Furthermore, in the past few years, interest in characterizing *in vivo* methionine oxidation has been revived^23^,^24^. Proteome-wide studies of *in vivo* methionine oxidation have led to the identification of a large number of cellular proteins that are targets of oxidative stress. Even more importantly, these proteomic efforts have allowed researchers to pinpoint the exact sites of oxidation on the target proteins. These data do not support random methionine oxidation and may indicate that not all accessible methionine residues in proteins are equally amenable to oxidation^23^. Nevertheless, despite the wealth of data provided by these studies and the current awareness regarding the relevant role of methionine residues, a number of key questions remain unresolved. For instance, if methionine oxidation is not a random process, what causes a given methionine residue to be resistant to oxidants while other residues are sensitive? In the current work, we have addressed this question with the aim of providing a better understanding of the factors that influence the likelihood of different methionine sites to become oxidized. We have found that the interaction of the methionine sulphur atom with the aryl moiety of aromatic amino acids is a key determinant that decreases the reactivity against peroxides. In addition, by taking advantage of this finding, we provide algorithms that allow accurate prediction of the redox status of a given methionine residue.

## Results

### The methionine abundance in H_2_O_2_-sensitive proteins is higher than that expected by chance

**Figure 1:**
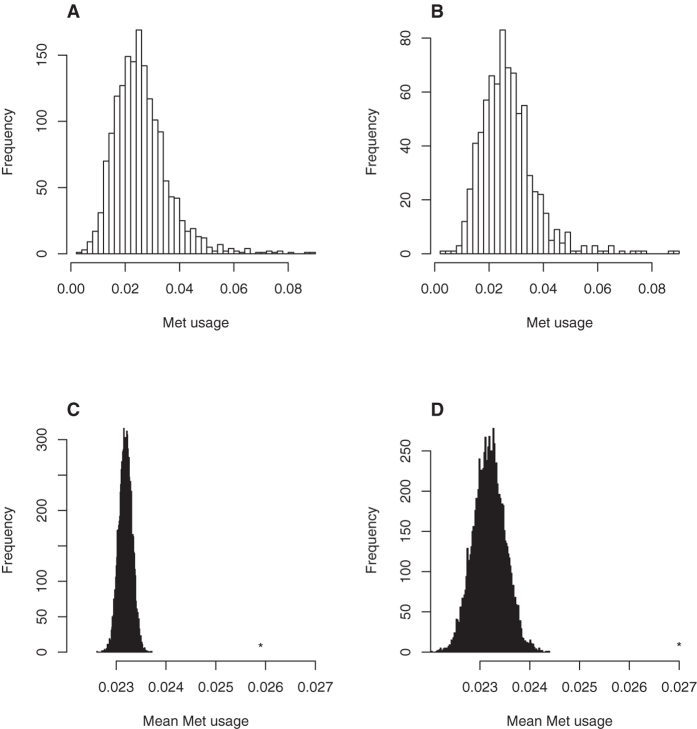
Oxidation-sensitive proteins are enriched in methionines. (**A**) Methionine frequency in the group of H_2_O_2_-sensitive proteins (1646 proteins, methionine usage: 0.0259 ± 0.0100 [mean ± standard deviation]). (**B**) Methionine frequency in the subset of highly H_2_O_2_-sensitive proteins (774 proteins, methionine usage: 0.0270 ± 0.0100 [mean ± standard deviation]). (**C**) Empirical distribution of mean methionine content for 10,000 random samples from the human proteome. For each sample, the mean methionine content was obtained by averaging across 1646 proteins randomly chosen from the human proteome. The position of the mean value computed for the set of H_2_O_2_-sensitive proteins is indicated with the symbol *. (**D**) Same as described in (**C**), but each sample was formed by 774 randomly selected proteins. The position of the mean methionine content value computed for the set of highly H_2_O_2_-sensitive proteins is again marked by *.

In previous research by Ghesquière and coworkers, over 2000 oxidation-sensitive methionines in more than 1600 different proteins from human Jurkat cells subjected to H_2_O_2_ stress were detected. These authors also quantified the degree of oxidation for each sensitive methionine^23^. Herein, we refer to the set of MetO-containing proteins as ‘H_2_O_2_-sensitive proteins’, and the subset of proteins containing over 20% oxidized methionines is referred to as ‘highly H_2_O_2_-sensitive proteins’. We started by questioning whether proteins detected as being oxidized *in vivo* are enriched in methionyl residues. In other words, does the methionine content of a protein influence the probability of its oxidation? A negative answer to this question would argue in favour of selective methionine oxidation mechanisms^25^. By contrast, a positive response would lend support to the hypothesis that methionine residues have a ROS scavenging function^26^, without invalidating a potential role for a subset of methionine residues in cellular signalling. Figure 1 shows the distribution of methionine content in the complete set of H_2_O_2_-sensitive proteins (Fig. 1A) and in the subset of highly H_2_O_2_-sensitive proteins (Fig. 1B). In both cases, the computed means (0.0259 and 0.0270, respectively) were greater than 0.0230, which is the average methionine usage reported for human proteins^27^. To assess how significant the observed differences are, we carried out bootstrapping analyses as described in the methodological section. We found that the higher methionine abundance observed in the set of H_2_O_2_-sensitive proteins (Fig. 1C) and in the subset of highly H_2_O_2_-sensitive proteins (Fig. 1D), could not be explained by sampling biases (p-values < 10^−4^). However, the biological relevance of these differences, if any, must be established.

### Differential sequence environments of resistant and sensitive methionines

In the previous section, we concluded that MetO-containing proteins tend to be enriched in methionines. Although this observation argues in favour of the anti-oxidant role postulated for methionines, it does not imply that all protein-bound methionines are equally prone to oxidation. In fact, it has been reported that methionine sulphoxides are preferentially found within a polar environment^23^, suggesting a non-random distribution pattern. However, because this study compared the frequencies of the neighbouring amino acids around the oxidized methionine with those of the theoretical human proteome, it cannot be ruled out that the observed associations reflect the general preferences of methionines, regardless of their reactivity towards oxidants. This is particularly relevant, given the existence of general short-range regularities in the primary structure of proteins. Indeed, it has been shown that every amino acid has a characteristic sequence environment within a ±10-residue distance^28^,^29^,^30^. Therefore, to define a consensus sequence pattern for oxidation-prone methionine sites, the neighbouring preferences of these sites must be compared to those of oxidation-resistant methionines. Hence, the first question to ask is whether the sequence environments of oxidation-prone and oxidation-resistant methionines are distinguishable. To address this question, we formulated the following null hypothesis: the normalized abundance of a given residue *i* in the vicinity (position *j*) of oxidation-prone methionines is the same as the normalized abundance of that residue in the neighbourhood of oxidation-resistant methionines. In other words, the standardized difference of the normalized abundances, Z_ij_, is a normally distributed random variable centred at 0. Thus, Z_ij_ positive values that deviate significantly from zero should be interpreted as follows: the amino acid *i* appears more often in the neighbourhood (at position *j*) of oxidation-prone methionines. Conversely, negative Z_ij_ values indicate a preference for the amino acid *i* to be located close to oxidation-resistant methionine (see Methods for details). Because 20 amino acids at 20 positions have been analysed, we have computed 400 values for the Z_ij_ variable. If the average occurrences of the various amino acids were the same in the neighbourhood of both types of methionine residues, then according to a typified normal distribution, fewer than 18 out of the 400 values would be expected outside the [−2, +2] interval, and only 1 would be expected outside the [−3, +3] interval. However, 101 and 48 values fell outside the [−2, +2] and [−3, 3] intervals, respectively. In fact, we observed two values outside the interval [−7, +7]. These observations allow us to conclude with very high confidence (p-value < 10^−23^) that oxidation-prone and oxidation-resistant methionines have different sequence environments.

**Figure 2:**
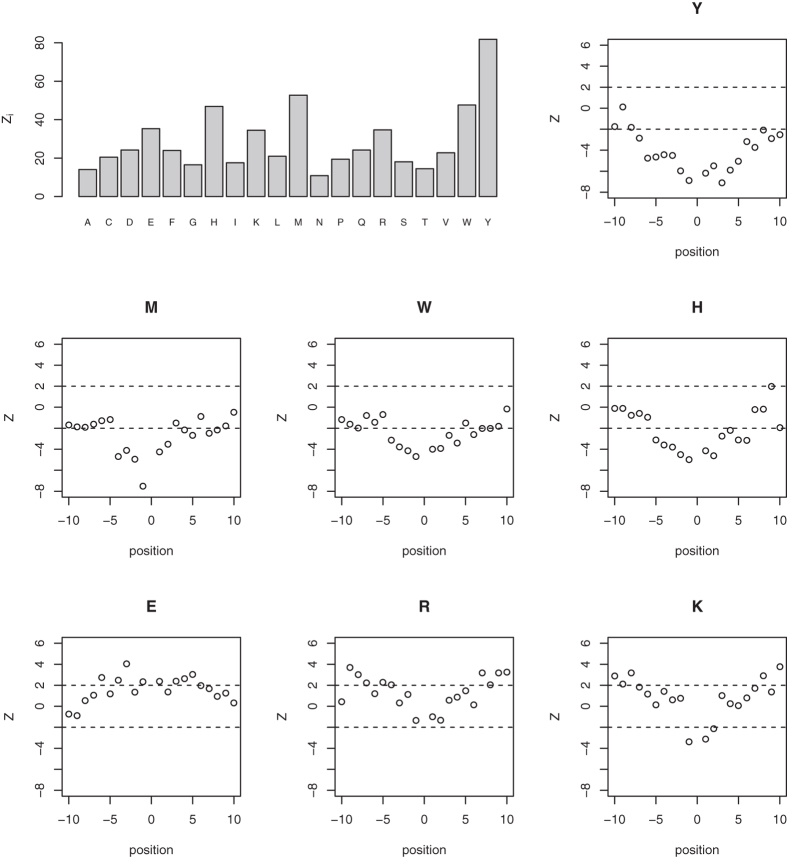
Differential sequence environment of oxidation-resistant and oxidation-prone methionines. The frequencies of amino acids flanking each oxidized methionine were recorded. This operation was repeated to compute the frequencies around non-oxidized methionine residues. The differences between these two frequency sets were used to determine the standard score, Z_ij_, according to the equation 1 from the text, where *i* indicates the amino acid whose frequency is being analysed and *j* is the position around the central methionine at which the frequency is being computed. A standard score that is much greater than zero or much less than zero indicates that amino acid *i* is overrepresented or underrepresented at position *j*, respectively, in an environment of oxidation-prone methionines with respect to an environment of oxidation-resistant methionines. The bar chart (upper left corner) represents the sum of the absolute values of the standard score for each amino acid, considering the 20 positions around the central methionine (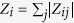

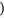
. At each position, the standard scores for the seven amino acids contributing more strongly to a differential environment (Y, M, W, H, E, R and K) were plotted.

It is remarkable that an oxidation-prone environment is defined by underrepresented amino acids rather than by overrepresented amino acids, as indicated by the fact that 77% of the standard scores that fell outside the [−3, +3] interval were negative. We next focused our attention on the relative importance of the different amino acids in determining an oxidation-prone environment. As shown in Fig. 2, there are 7 amino acids that stand out among the rest. The aromatic residues tyrosine and tryptophan together with methionine make the strongest contribution. These three amino acids are underrepresented in the proximity of oxidized methionines. However, amino acids with ionizable side chains, with the exception of aspartate and cysteine, also make a significant contribution to the environment. Thus, while the acidic amino acid glutamate is most often found close to oxidation-prone methionines, the basic amino acids histidine and lysine are not often found near the central position. However, further away from the central oxidation-prone methionine, both lysine and arginine are overrepresented (Fig. 2). In summary, from the most distant position towards the central methionine, the charge tends to change from positive to negative, and the probability of finding an aromatic residue drops significantly.

### Computational intelligence and oxidation site prediction using primary structure

**Figure 3:**
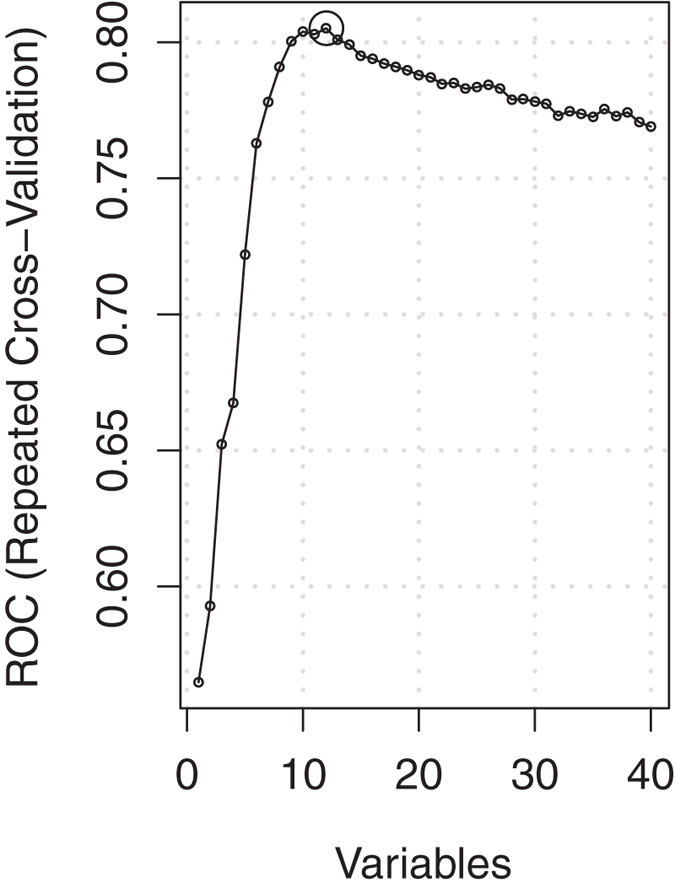
Variables related to basic and aromatic amino acids can be useful for accurate prediction of oxidation sites. The plot shows the relationship between the number of variables and the resampled estimate of the area under the ROC curve for 10-fold cross-validation with 5 repetitions (50 resamplings), obtained from feature selection with RFE and RF-wrappers. The best performance (indicated by the marked point in the plot) was obtained when the following twelve variables were considered: NT_M, CT_M, NT_Y, NT_K, CT_Y, NT_R, CT_K, CT_R, CT_H, CT_W, NT_W and CT_F, where NT_X and CT_X stand for the location of the amino acid X with respect to the methionine being analysed (i.e., “NT” indicates that amino acid X is N-terminal to the methionine being analysed, and “CT” indicates that amino acid X is C-terminal to the methionine being analysed).

The results presented thus far suggest that methionines prone to oxidation (those that appear as MetO *in vivo*) may share certain features that may be absent in their oxidation-resistant counterparts. If this is indeed the case, then it should be possible to design predictive models via machine learning that are aimed at predicting oxidation sites using only information about the primary structure of the protein. Table 1 shows the results from a random forest (RF) predictive model of oxidation sites for both the training dataset (10-fold cross-validation with 5 repetitions) and the testing set. Feature selection using recursive feature elimination (RFE) gave the following 12 most relevant features (Fig. 3): NT_M, CT_M, NT_Y, NT_K, CT_Y, NT_R, CT_K, CT_R, CT_H, CT_W, NT_W and CT_F, where NT_X and CT_X stand for the location of the amino acid X with respect to the methionine being analysed (i.e., “NT” indicates that amino acid X is N-terminal to the methionine being analysed, and “CT” indicates that amino acid X is C-terminal to the methionine being analysed). In accordance with the results presented in the preceding section, aside from methionine itself, basic and aromatic amino acids seem to be determinant. These 12 variables were finally used to train an RF model, whose performance is also summarized in Table 1.

### The S-aromatic motif is a key determinant of methionine redox status

**Figure 4:**
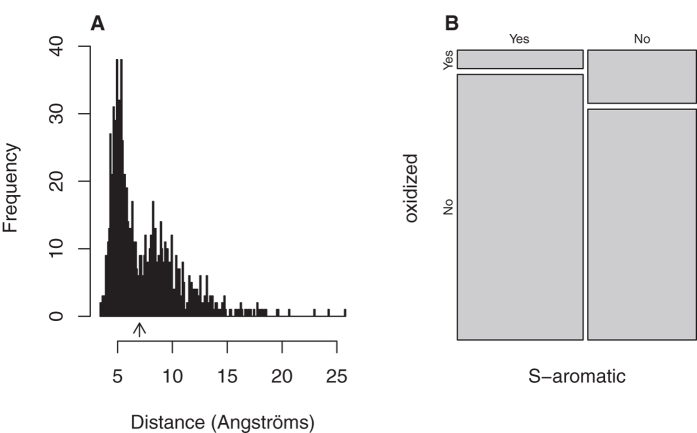
Methionines forming part of an S-aromatic motif are less likely to be oxidized. (**A**) For each of the 1118 methionines present in the set of highly H_2_O_2_-sensitive proteins with known structures, the distance from the sulphur atom to the geometric centre of the aryl moiety of any aromatic residue was computed. The distribution of such distances was plotted and shows a characteristic bimodal distribution. Any methionine sulphur atom within 7 Å (arrow) of the aromatic ring centre from any aromatic amino acid was considered to be part of an S-aromatic motif. (**B**) The interrelationship between the propensity to form S-aromatic motifs and the propensity to be oxidized is shown as a mosaic plot, which allows us to easily see that the proportion of S-aromatic motifs that are oxidized is very different from the proportion of S-aromatic motifs that are not oxidized. The significance of the difference (p-value = 4 × 10^−10^) was assessed using Fisher’s exact probability test.

Above, we have presented evidence indicating an important role for aromatic residues in the conformation of different sequence environments as well as in the prediction of oxidation sites involving methionines (see selected features). One interesting characteristic of methionyl residues, which has been largely overlooked, is their propensity to interact with the aromatic side chains of residues such as phenylalanine, tyrosine and tryptophan^31^, contributing to the stabilization of protein structure^32^. Together, these observations prompted us to investigate the potential role of methionine-aromatic motifs as determinants of methionine oxidation. To this end, among the most highly H_2_O_2_-sensitive proteins, we selected those with known structures. This collection, comprising 127 different polypeptides, includes 1118 methionyl residues, 136 of which have been detected as MetO *in vivo*. We first assessed whether this assembled data set could be taken as a representative sample of the more than 80,000 known protein structures with respect to the sulphur-aromatic interaction. In agreement with a previous large-scale bioinformatics study^32^, we observed enrichment of methionine sulphur atoms within 7 Å of any aromatic group, with a prominent peak corresponding to a sulphur-aromatic separation of 5 Å and a second peak at approximately 8 Å (Fig. 4A). This bimodal distribution strongly suggests that our collection of 1118 methionines represents a fair and unbiased sample. Therefore, using the same criterion of other authors, we considered the sulphur-aromatic interaction to be any methionine sulphur atom within 7 Å of the aromatic ring centre of mass based on the first minimum in the distribution (Fig. 4A, arrow). We next tested the null hypothesis that the tendency of a given methionine residue to form an S-aromatic bond is independent of the propensity of such a residue to appear oxidized after an oxidative insult. To test this hypothesis, we used Fisher’s exact probability test (Fig. 4B). Although more than half of the analysed methionines were involved in S-aromatic motifs, this proportion fell below 29% when the analysed methionines were restricted to those detected as oxidized *in vivo*. Under the conditions of the null hypothesis, the probability that the 136 oxidation-prone residues would be so unevenly distributed between the categories of “forming S-aromatic motifs” and “not forming S-aromatic motifs” was calculated to be as low as 4.0 × 10^−10^. Therefore, we rejected the null hypothesis and concluded that methionines taking part in S-aromatic interactions are less likely to oxidize.

### Relationships among oxidation, accessibility and S-aromatic interaction

**Figure 5:**
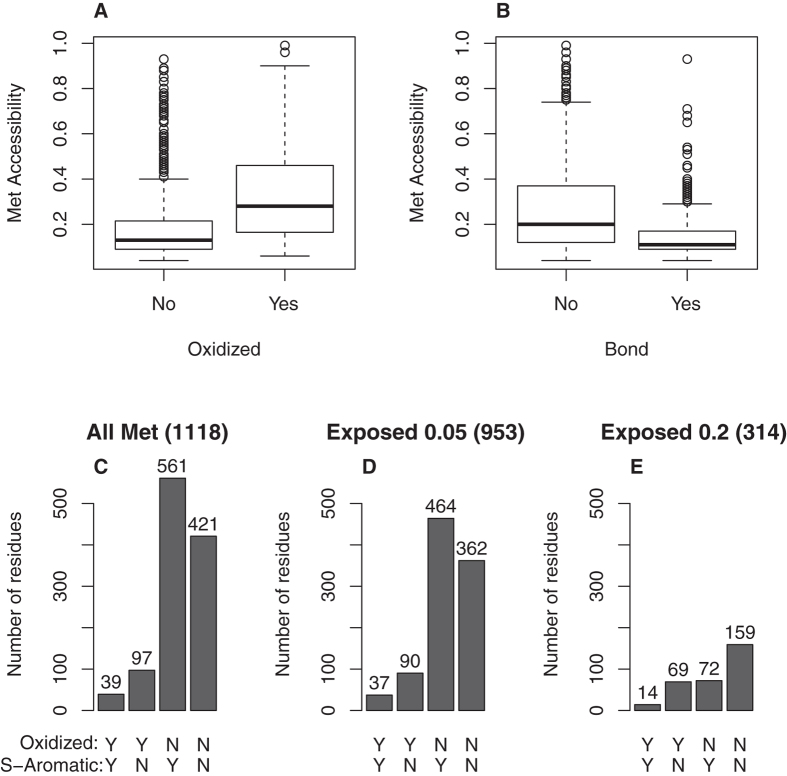
Relationships among methionine oxidation, methionine accessibility and methionine participation in S-aromatic motifs. (**A**) Box plot of accessibility of oxidized and non-oxidized methionines. (**B**) Box plot of accessibility of methionines that form and do not form S-aromatic motifs. (**C**–**E**). Bar plots showing the number of oxidized methionines that participate in S-aromatic motifs (YY), oxidized methionines that do not participate in S-aromatic motifs (YN), non-oxidized methionines involved in S-aromatic motifs (NY) and non-oxidized methionines that do not form S-aromatic motifs (NN). (**C**) shows the results for all methionines, while (**D,E**) show the results obtained after excluding buried methionines according to two accessibility criteria (0.05 and 0.20 accessibility). In each case, the number of methionine residues analysed is indicated in the brackets.

As mentioned in the Introduction, the relationship between the solvent-accessible area of a methionine residue and its vulnerability to oxidants remains somewhat controversial. Thus, although most authors accept that exposed residues are readily oxidized, a recent study by Vandermarliere *et al.* has failed to find a correlation between the degree of oxidation and the relative solvent-accessible surface^9^. From this lack of correlation, the authors concluded that there is no link between buried or exposed residues and the degree of oxidation. Herein, we have addressed this issue using a different approach. Using a data set consisting of highly H_2_O_2_-sensitive proteins, the methionine residues were categorized as “exposed” or “buried” according to accessibility criteria, as explained in the Methods section. Using Fisher’s exact test, we refuted the null hypothesis that oxidized methionines are equally distributed between the protein surface and its interior (p-value = 3 × 10^−16^); thus, we postulate that buried methionines are less likely to appear oxidized. Because aromatic residues may interact preferentially with buried methionines (Fig. 5A,B), our next goal was to elucidate whether the relationship we have established between methionine oxidation and methionine participation in S-aromatic interactions might be accounted for by a confounding factor such as residue accessibility. To this end, we re-addressed the distribution of oxidized methionines that form S-aromatic motifs and those that do not form S-aromatic motifs after excluding buried methionines from the analysis. Using two extreme accessibility criteria (see Methods), the results clearly indicate the same conclusion: the proportion of oxidation-prone methionines involved in S-aromatic motifs is much less than that expected by chance (Fig. 5C–E). Thus, the affirmation that methionines that interact with aromatic amino acids are significantly underrepresented among oxidized methionines, which was reached by analysing all methionines, still holds when the analysis is restricted to exposed methionines (p-values = 10^−8^ and 0.014 for accessibility criteria of 5% and 20%, respectively).

### Reactivity versus specificity

Thus far, we have described the remarkably low propensity of methionine moieties from S-aromatic motifs to appear as MetO. However, it should be noted that the methionine sulfoxide proteome used in these analyses represents a steady-state situation, in which oxidation after hydrogen peroxide challenge is balanced by reduction catalysed by methionine sulfoxide reductases (Msrs). Therefore, two alternative hypotheses may explain our results. On the one hand, the interaction of methionine with the ring of an aromatic residue may decrease the ability of the sulphur atom to react with H_2_O_2_. On the other hand, given the biological relevance of the S-aromatic interaction^32^,^33^, it may be possible that Msrs have evolved to preferentially recognize the sequence environment of these structural motifs and to repair them when they become oxidized. If the reactivity hypothesis is correct, then we can predict that the methionine residues involved in S-aromatic interactions from a protein exposed to H_2_O_2_ will be less likely to be oxidized *in vitro* (in the absence of Msrs). Thus, we next focused on testing such a prediction. To this end, we took advantage of a number of studies in which the reactivities of protein pharmaceutical-derived methionine residues against H_2_O_2_ have been reported (Table 2). Because the reaction conditions (temperature, pH, solutes, reagent concentrations, etc.) were not comparable from one study to another, the methionine reactivities reported within each single study were assigned to one of two categories: ‘low reactivity’ and ‘high reactivity’. Once each methionine was assigned to one of these two categories according to its empirical reactivity, we proceeded to reclassify the methionines following a simple predictive rule: methionines involved in S-aromatic motifs were predicted to belong to the ‘low-reactivity’ group, and all other methionines were assigned to the ‘high-reactivity’ group. The performance of this simple classifier is shown in the form of a confusion matrix (Table 3). From a total of 35 methionines, 30 were correctly classified, accounting for an accuracy of 85.7%. Furthermore, the sensitivity (true positive rate, 85%) and specificity (true negative rate, 86.7%) were equally high and well balanced between them. This highly significant enrichment of S-aromatic motifs within the low-reactivity group (p-value = 3 × 10^−5^), allows us to conclude that the involved methionines exhibit a much lower reactivity than their non-interacting counterparts. In addition, this property provides a simple, accurate and reliable method to predict the vulnerability of a given methionine residue to oxidation *in vitro.*

At this point, we questioned whether the relationship between methionine reactivity and S-aromatic bonds that we described above for human proteins could be extended to prokaryotic proteins. Although providing a statistically robust answer to this question was beyond the scope of the current study, we wanted to assess the performance of our prediction rule (highly oxidizable residues ⇔ S-aromatic motifs are absent) using an *Escherichia coli* enzyme, glutamine synthetase, with well-documented reactivity to H_2_O_2_ *in vitro*^26^. The results of this analysis are shown in Table 4. Our simple rule was able to correctly assign the redox status of 13 out of 15 methionyl residues (p-value = 0.003) that have been empirically well characterized.

## Discussion

Oxidation of protein-bound methionines can be reversed by reducing enzymes termed methionine sulfoxide reductases. Despite the widespread belief of the importance of these enzymes, their precise physiological functions remain elusive. One hypothesis is that these enzymes fulfil a repair function by reducing oxidized methionines that are essential for protein stability/function^34^. A second hypothesis postulates a scavenger role for Msrs. According to this hypothesis, highly reactive surface-exposed methionine residues can serve as endogenous antioxidants, acting as reactive oxygen species (ROS) sink sites^26^. A third emerging hypothesis states that methionine oxidation provides the cell with information on its oxidative state, playing an important role in signalling^35^,^36^,^37^,^38^.

Thus, the question of whether methionine oxidation is simply an unavoidable consequence of oxidative stress or a protective mechanism against oxidative damage has given rise to an ongoing debate with far-reaching consequences. The available evidence suggests that the longer the lifespan of a species, the lower its tissue protein methionine content^39^,^40^. This observation, however, has been interpreted in two opposite ways. On one side, it has been argued that the lower abundance of methionyl residues in proteins from long-lived species confers a decreased vulnerability to oxidative damage, which, in turn, may contribute to the longer lifespans of these species^40^,^41^,^42^. In contrast to this interpretation, we have argued that if methionine residues serve as a ROS sink, then proteins from animals subjected to high levels of oxidative stress should accumulate methionine more effectively than orthologous proteins from species exposed to lower levels of oxidative stress. In other words, the high methionine content observed in short-lived species, which are known to produce ROS at higher rates^43^, may represent an adaptive response driven by a high selection pressure favouring the accumulation of methionine residues in proteins^39^,^44^.

The observation that the set of proteins containing oxidized methionines is enriched in methionine residues supports the hypothesis that methionines serve as ROS scavengers; however, it does not invalidate the possibility that a few particular residues may be involved in redox signalling. In this respect, it is notable that proteins with very low methionine usage can be found in both human and plant methionine sulfoxide proteomes^23^,^25^,^35^. On the other hand, the potential role of methionines as ROS scavengers does not imply in any way that all protein-bound methionines are equally prone to oxidation. In fact, we found differences between oxidation-prone and oxidation-resistant methionine sequence environments (Fig. 2). The former can be defined in statistical terms as environments in which glutamate is overrepresented, whereas basic and aromatic residues are clearly underrepresented. Furthermore, features related to basic and aromatic amino acids allow accurate prediction of oxidation sites (Table 1 and Fig. 3). We focused our attention on aromatic residues because they are known to interact with sulphur atoms. The energy associated with a single sulphur-aromatic interaction is comparable to that of a single salt bridge, but the former can occur at longer distances^32^,^33^. Given the prevalence of methionine-aromatic interactions in known protein structures and the observed underrepresentation of aromatic residues in areas close to oxidation-prone methionine residues, we decided to address whether oxidation sites exhibited a distinguishable propensity to participate in the formation of S-aromatic motifs. Indeed, this was the case, as only 39 out of 600 methionine-aromatic motifs were susceptible to oxidation (Fig. 4B). The probability of observing such an uneven proportion, under the assumption that the tendency of a given methionine to interact with an aromatic residue is independent of the propensity of such a residue to be oxidized, was determined to be exceedingly low (4 × 10^−10^). There is no doubt that for a given methionine residue, its propensity to participate in a S-aromatic bond and its propensity to become oxidized are not independent properties. However, the lack of independence does not necessarily mean a causal relationship. We could not rule out the possibility that methionine-forming bonds were less vulnerable to oxidation simply because they were less accessible to solvent. In fact, the distribution of the accessibility computed for methionines forming S-aromatic interactions mirrored that of oxidation-resistant methionines (Fig. 5A,B), which might indicate that the accessibility of the methionine to oxidants could be a confounding factor, and the observed relationship between S-aromatic-forming residues and oxidation-resistant methionines might be spurious. However, this *a priori* plausible scenario can be disregarded because exposed methionines interacting with aromatic residues also showed a low propensity to be oxidized. Furthermore, this low propensity to appear oxidized can unequivocally be interpreted in terms of the decreased reactivity of the involved methionine, as strongly suggested by the link between low-reactivity and S-aromatic motifs, in proteins whose reactivities were assayed *in vitro* (Tables 3 and 4) in the absence of reducing enzymes.

The statistical analyses of interatomic distances described above allow us to confidently conclude that methionine residues close to aromatic rings in the tertiary structure are less likely to be oxidized, a phenomenon that has remained unnoticed until now. However, methionines that are located close to tyrosine and tryptophan in the primary structure also appeared to be oxidation-resistant. Nonetheless, a link between these two findings should not be taken for granted because residues that are distant from each other in the primary structure can often interact closely with each other due to protein folding. In fact, when we examined the distances between the aromatic residue and the methionine in all of the S-aromatic motifs analysed in the current study, we observed that the interacting aromatic residue was located outside of the sequence environment of the methionine in many cases (Table 5). Furthermore, although phenylalanine is the aromatic amino acid that most often has a role in S-aromatic motifs, it does not contribute to the differential sequence environments of resistant and sensitive methionines (Fig. 2). Thus, the question of why tyrosine and, to a lesser extent, tryptophan are much more abundant near non-oxidized methionines compared with oxidized methionines deserves further investigation in the future. Nevertheless, the finding that S-aromatic motifs formed exclusively by phenylalanine were still less likely to be oxidized (data not shown, p-value < 3 × 10^−8^, Fisher’s exact probability test) strongly supports our conclusion that the S-aromatic motif is a relevant determinant of methionine oxidation.

To the best of our knowledge, we are the first to identify the methionine-aromatic motif as a structural determinant of methionine oxidation. These motifs alone are able to influence the reactivity of the methionine moiety. Our results also highlight the interrelated, and somewhat complementary, roles of solvent accessibility and S-aromatic motifs in determining the vulnerability of methionines towards oxidation, thus clarifying the complex correlation between structural properties and methionine oxidation. In the future, as our ability to predict oxidation sites improves, it will likely be relatively easy to engineer a protein to resist oxidative destabilization. In addition, a better understanding of the structural determinants of methionine oxidation should also facilitate evaluations of the physiological significance of reversible modification of a given methionine residue. Therefore, further work is needed to provide a comprehensive picture of methionine oxidation, which will provide a rationale for developing strategies to control oxidation.

## Methods

### Datasets

Data regarding methionine peptides that were oxidized in Jurkat cells subjected to H_2_O_2_ stress were taken from Table S1 in the supplementary material of Ghesquière *et al.*^23^. This set was further curated to exclude protein entries that have recently been deleted from UniProt (http://www.uniprot.org). The resulting data set, which is referred to as ‘H_2_O_2_-sensitive proteins’, comprises 1646 different proteins accounting for 2616 methionine sulphoxides. A subset of this collection, comprising 774 proteins that exhibit extensive oxidation (equal or greater than 20% oxidation), was named ‘highly H_2_O_2_-sensitive proteins’.

### Empirical distributions of methionine content

Ten thousand random samples, each including 1646 human proteins, were collected from UniProt. For each sample, the mean value of methionine content was obtained by averaging across all the randomly chosen proteins. This collection of 10^4^ mean values was used to plot the distribution of mean values for methionine abundance and contrast the mean computed for the 1646 H_2_O_2_-sensitive proteins. It should be noted that the N-terminal initiating methionine residue was removed from the sequences analysed. The same procedures were repeated for the subset of highly H_2_O_2_-sensitive proteins, although random samples of 774 human proteins were used. These empirical distributions were used to provide statistical support for our comparative methionine content analyses.

### Comparison of sequence environments

For each oxidation site, that is, for each methionine residue that was detected as MetO, the frequency of flanking amino acids at each position from –10 to +10 relative to the central methionine was recorded. Only methionyl residues with ten or more neighbours in both directions were included in this analysis. Thus, we obtained a square matrix of order 20, {f_ij_^MetO^}, where f_ij_^MetO^ is the computed relative frequency for amino acid i at position j. For each oxidation site, a non-oxidized methionine within the same protein was randomly chosen and subjected to the same frequency analysis to derive the corresponding frequency matrix, {f_ij_^Met^}. Because we were interested in detecting differences between the sequence environments of oxidation-prone and oxidation-resistant methionines, we formulated the null hypothesis that the difference in these two frequency matrices yields the zero matrix. To contrast this hypothesis, a new matrix, {Z_ij_}, accounting for the standardized difference in frequencies, was computed according to equation (1):




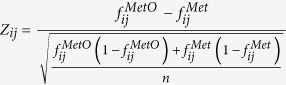




where n is the total number of oxidation sites being analysed. Under the null hypothesis condition, this typified variable must follow a normal distribution with mean 0 and variance 1. Therefore, standard scores (Z_ij_) that are extremely greater than or extremely less than 0 may indicate a preference for (positive sign) or disfavour of (negative sign) the residue i around oxidation sites.

### Computational intelligence approach to oxidation site prediction

Using proteins from the highly H_2_O_2_-sensitive group, a benchmark dataset composed of 908 oxidation-prone methionines and 830 oxidation-resistant methionines was established. For each methionine from this dataset, a collection of 40 ‘distance’ variables was extracted. Given an amino acid, e.g., alanine, we searched in the proximity of the methionine of interest for the closest alanine residue towards the N-terminus and for the closest alanine residue towards the C-terminus. Once these alanine residues were found in the primary structure, we counted the number of residues away from the methionine being analysed (NT_A and CT_A variables). This operation was repeated for each of the 20 proteinogenic amino acids to obtain a set of 40 characteristics used as predictors of methionine oxidation. For missing values due to the absence of any particular amino acid either towards the N-terminus or the C-terminus, the protein length was taken as a default value. To fit predictive models, this dataset was divided into two independent groups: 75% patterns for training and 25% for testing. Among the classification techniques explored, random forest (RF) showed the best performance. RFs^45^ are ensemble machine learning methods for classification that function by constructing a large pool of decision trees (we fixed the number of trees to 1,000) on bootstrapped training samples. Thus, this method combines many decision trees to make a prediction: for a given input pattern (i.e., a 40-feature vector), the RF’s output (oxidized/not-oxidized) is simply the mode of the outputs given by the trees in the pool. Basically, to train an RF, each one of the decision trees is built by performing recursive binary splits of the predictor space to obtain a pool of non-overlapping regions that minimizes the *Gini index*, i.e., the total variance across the two classes. At each split, from the full set of 40 predictors, *m* predictors are chosen at random as candidates. Typically, *m* is equal to the truncated square root of the total number of predictors^46^ (6 out of the 40, for our data). To estimate the efficacy of the RF model across the training set, performance measures such as the area under the ROC curve (AUC), accuracy, sensitivity and specificity were assessed using the out-of-bag samples (i.e., samples excluded by the bootstrap iterations) for 10-fold cross-validation with 5 repetitions (50 re-samplings). The entire training set was used to fit a final model, and its performance was measured on the testing set. Finally, to assess the importance of each predictor and select characteristics with the highest predictive power, a feature selection algorithm, known as recursive feature elimination (RFE), was arranged and applied on the training set. The R packages *randomForest*^47^ and *caret*^48^ were used for these analyses.

### Sulphur to aromatic ring distances

Using PDB cross-references from UniProt, we collected a list of PDB identifiers for proteins belonging to the highly H_2_O_2_-sensitive group. In general, because many proteins were homooligomers, most crystal structures yielded a large number of duplicated observations, which were searched for and eliminated using an R script. Eventually, after removing redundancy and filtering out low-quality structures (for instance, those in which the target methionine did not appear to be resolved), we assembled a collection of 127 unique polypeptides of known structure containing 1118 methionyl residues, 136 of which were oxidation-prone. For each methionine, the distance from the sulphur atom to the geometric centre of the aryl moiety of any aromatic residue was computed with the aid of an *ad doc* R script that relies on the package bio3d^49^. Based on a previously established criterion^32^, we considered any methionine sulphur atom within 7 Å of the aromatic ring to be an S-aromatic motif. For each of the 1118 methionines, relevant information such as its redox status, the corresponding PDB identifier, the positions within the structure of both the analysed methionine and the closest aromatic residues (as well as their distances in Å) are available upon request.

### Accessibility

The solvent-accessible surface area (SASA) of each methionine residue was computed using the POPS program^50^. This software also provides the accessibility, which is defined as the fraction of the residue surface that is exposed to solvent. Accessibility is commonly used to classify residues as exposed or buried, depending on whether their accessibilities exceed or do not exceed an established threshold, respectively. In the literature, different thresholds have been employed as criteria^9^,^51^,^52^. Thus, to strengthen any conclusion derived from analyses involving accessibility, we determined the robustness of our results by using two extreme threshold values: 5% and 20%.

### Therapeutic protein oxidation

We searched the literature to collect data on the reactivity of methionyl residues from protein pharmaceuticals. We gathered data for 8 proteins that satisfy the following requirements: i) the protein should contain at least two methionines with different reactivities; ii) the kinetics of *in vitro* oxidation of these residues with H_2_O_2_ must be reported in the literature; and iii) the structure of the protein should be known, and it must be available in the PDB. For each protein, the reactivities of its methionines were ordered from low to high. Residues showing reactivities lower than the median were labelled as ‘low reactivity’ residues, and residues with reactivities above the median were labelled as ‘high reactivity’ residues. Methionines with reactivities equal to the median were sorted into the group containing the residue with a reactivity closest to the median.

## Additional Information

**How to cite this article**: Aledo, J.C. *et al.* Sulphur Atoms from Methionines Interacting with Aromatic Residues Are Less Prone to Oxidation. *Sci. Rep.* **5**, 16955; doi: 10.1038/srep16955 (2015).

## Figures and Tables

**Table 1:** Performance of the RF predictive models.

		AUC	Accuracy	Sensitivity	Specificity
RF	Training	0.7725	0.7092	0.7927	0.6180
	Testing	0.7649	0.7005	0.8018	0.5894
RF-RFE	Training	0.8051	0.7376	0.7962	0.6735
	Testing	0.7573	0.6912	0.7709	0.6039

**Table 2:** *In vitro* oxidation of methionine residues from therapeutic proteins.

Protein	PDB	Low	High	TP	TN	FP	FN	Ref
Granulocyte Colony-Stimulating Factor	1CD9	122	127,138	1	2	0	0	8
Alpha1-Antitrypsin	1HP7	63,220,221,242,374,385	226,351,358	5	3	0	1	7
Prion Protein	1B10	154,206,213	129,134	2	2	0	1	53
IgG1-Fc	1FC1	358,428	252	2	1	0	0	54
Stem Cell Factor	1EXZ	36,48	27	2	1	0	0	55
Coagulation Factor VIIa	1QFK	327,391	298,306	2	1	1	0	56
Growth Hormone	1HGU	170	14,125	1	2	0	0	57
Chorionic Somatomammotropin	1Z7C	14,96,170	125,179	2	1	1	1	57

The columns *Low* and *High* provide the positions of the methionines for which low and high reactivity, respectively, have been experimentally established. Whenever a methionine participated in an S-aromatic motif, it was predicted to have a low reactivity. Otherwise, the methionine was predicted to have a high reactivity. The numbers of true positive (TP), true negative (TN), false positive (FP) and false negative (FN) results are shown.

**Table 3:** Confusion matrix for the model: S-aromatic ⇔ Low reactivity.

		S-aromatic	
		Yes	No
Reactivity	Low	17	3
	High	2	13

**Table 4:**
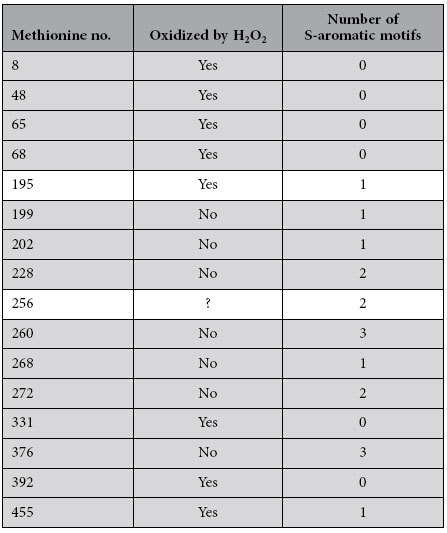
Relationship between methionine reactivity and S-aromatic motifs in a prokaryotic protein.

Data related to the susceptibility of methionine residues in bacterial glutamine synthetase to oxidation were taken from a seminal work by Levine and coworkers^26^. Using the atomic coordinates of this enzyme (PDB ID: 2GLS), we computed the number of S-aromatic motifs formed by each methionine residue. We defined a random variable, X, as the number of correct guesses. A guess was considered correct when an oxidized methionine did not participate in S-aromatic motifs or when a non-oxidized methionine formed at least one S-aromatic motif (shaded rows). Under the null hypothesis of random guessing, this random variable should be distributed according to a binomial distribution with p ≅ 0.5 and n = 15. Because we observed X = 13, the p-value for this observation can be calculated as P[X ≥ 13] = 0.003.

**Table 5:** Linear distances between the methionine and the aromatic residue from S-aromatic motifs.

Aromatic residue	n	Median	Mean	Standard deviation
Phe	596	15	36.2	53.9
Tyr	412	13	43.3	81.6
Trp	96	26.5	65.3	93.8

For each methionine forming an S-aromatic motif, the linear distance to the interacting aromatic residue in the primary structure was computed. The median, mean and standard deviation of the number of residues between the methionine and the aromatic residue are shown. The number of aromatic amino acids interacting with methionine, n, is also indicated.
